# Pathophysiology of *Myopenia* in rheumatoid arthritis

**DOI:** 10.1038/s41413-025-00438-9

**Published:** 2025-06-16

**Authors:** Haiming Jin, Gang Wang, Qichen Lu, Jessica Rawlins, Junchun Chen, Saanya Kashyap, Oscar Charlesworth, Dan Xu, Lie Dai, Sipin Zhu, Jiake Xu

**Affiliations:** 1 Department of Orthopaedics, The Second Affiliated Hospital and Yuying Children’s Hospital of Wenzhou Medical University, Wenzhou, Zhejiang 325000 China; 2 School of Biomedical Sciences, The University of Western Australia, Perth, WA 6009 Australia; 3 Shenzhen University of Advanced Sciences, and Shenzhen Institutes of Advanced Sciences Chinese Academy of Sciences, Shenzhen, China; 4 Curtin School of Population Health/Curtin Medical School, Faculty of Health Sciences, Curtin University, Perth, Western Australia Australia; 5 Department of Medical Education, First Affiliated Hospital, Sun Yat-Sen University, Guangzhou, Guangdong China; 6 Department of Rheumatology and Immunology, Sun Yat-Sen Memorial Hospital, Sun Yat-Sen University, Guangzhou, Guangdong China

**Keywords:** Metabolism, Pathogenesis

## Abstract

Rheumatoid arthritis (RA) is a prevalent and debilitating inflammatory disease that significantly impairs functional capacity and quality of life. RA accelerates musculoskeletal aging, leading to complications such as muscle degeneration and *sarcopenia*. Recent research has identified *myopenia* as a condition of significant muscle loss associated with illness, distinct from the muscle wasting seen in other chronic diseases like cancer cachexia or heart failure. In RA, *myopenia* is characterized by muscle depletion without concurrent significant fat loss, and it can affect individuals of all ages. While inflammation plays a central role, it is not the sole factor contributing to the high incidence of muscle wasting in RA. In subsequent discussions, *secondary sarcopenia* will be considered alongside *myopenia*, as both involve muscle wasting decline primarily due to disease. This review summarizes recent findings on the impact of RA-related *myopenia* and *secondary sarcopenia* on functional capacity, explores its underlying mechanisms, and discusses contemporary strategies to mitigate the process of musculoskeletal aging in RA patients.

## Introduction

Rheumatoid arthritis (RA) is a chronic autoimmune disease characterized by erosive arthritis, accelerated musculoskeletal aging, and systemic organ involvement. It affects approximately 0.5% of adults globally.^1^ RA predominantly affects women between the ages of 25 and 45 but can occur in individuals of any age and gender, with the highest incidence typically observed in the sixth decade of life. RA manifestation prior to the age of 60 is classified as *young-onset RA (YORA)*, while onset after 60 is termed *elderly-onset RA (EORA)*.^2^ Current demographic trends reveal that the prevalence of *EORA* is significantly influenced by sex and ethnicity. In the United States, the prevalence ranges from 0.5% to 1%, increasing to 2% among those over 60 years of age.^3^ Conversely, in China, pooled prevalence estimates indicate that 0.37% of the population suffers from RA, with 25.7% classified as *EORA*.^4^ These trends underscore the importance of considering ethnic and geographic factors in managing of RA.

Within the RA context, muscle wasting, also known as *myopenia*, represents a critical area of concern. Recently introduced, *myopenia* describes substantial muscle loss due to illness at any age and is marked by reduced muscle function and increased rates of morbidity and mortality.^5^ *Myopenia* holds particular relevance in RA, where chronic inflammation and systemic involvement lead to significant muscle degradation. Although the exact mechanisms underlying *myopenia* in RA remain elusive, they are believed to involve a complex interplay of immunological and hormonal changes, coupled with cytokine activity, contributing to muscle wasting and premature muscular aging. Recent studies have highlighted additional factors such as *Oxidative stress, intermuscular adipose tissue (IMAT) accumulation, and insulin resistance*, all of which exacerbate muscle wasting and further complicate disease progression. Elevated levels of myostatin, a negative regulator of muscle growth, may contribute to muscle wasting or rheumatoid cachexia (RC).^6,7^ A deeper understanding of the pathophysiology of RA is essential for developing effective treatments that can mitigate musculoskeletal decline and enhance patient outcomes.

Current strategies for managing *myopenia* in RA focus on lifestyle enhancements and risk factor modification through adequate nutrition and regular physical activity.^8^ Furthermore, the. Early administration of *disease-modifying antirheumatic drugs (DMARDs)* is vital, as Treat-to-Target therapy has been shown to prevent radiological damage, reduce morbidity and mortality, and improve overall functional capacity.^9^ This review aims to highlight the clinical significance of *myopenia* in RA and explore various strategies to delay musculoskeletal aging. Key approaches include managing early inflammation through lifestyle modifications, pharmacological interventions and reducing *Oxidative stress* to aid muscle regeneration. By addressing these areas, this review seeks to provide valuable insights and propose comprehensive strategies for effectively managing muscle wasting in individuals with RA.

## Musculoskeletal aging in healthy individuals

Muscle remodeling naturally occurs with age, with muscle mass declining by 1%–2% annually after age 50, and accelerating significantly after age 70, particularly in men, who experience greater absolute and relative muscle loss.^10^ This is accompanied by increased intramuscular fat, muscle atrophy (especially in type IIa fibers, which affect strength and explosive power), reduced motor unit numbers, and impaired satellite cell function.

Aging also decreases muscle capillarization, affecting metabolic health and cardiovascular fitness.^11^ Inflammaging, marked by increased pro-inflammatory markers, further accelerates muscle degradation by impairing the muscle’s adaptive response to exercise.^12^ This remodeled muscle architecture significantly leads to reductions in muscle strength and power commonly observed in elderly individuals.^13,14^ Frailty, which marks an individual’s transition to disability, is characterized by symptoms such as fatigue and weakness, closely associated with muscle loss.^15^

Research conducted by Mitchell and colleagues indicates that after the age of 70, muscle loss occurs at a rate of 0.5%–1.0% annually with peak muscle mass decreasing by 4.7% per decade in men and 3.7% in women.^10^ This phenomenon is closely linked to age-related *sarcopenia*, which is characterized by a progressive decline in skeletal muscle mass and function. Muscle strength decreases by 10%–15% per decade until age 70, after which it accelerates to 25%–40% per decade.^15,16^ This decline is driven by both muscle mass loss and neuromuscular aging, including motor neuron loss, motor unit reduction, and neuromuscular junction degeneration.^17^

Frailty, characterized by reduced grip strength and slower gait speed, increases vulnerability to stressors and is more pronounced in women, who start with lower lean body mass, making them more susceptible to sarcopenia and its consequences, such as falls, disability, and mortality.^15^

Chronic conditions like cancer, diabetes, COPD, and heart failure exacerbate muscle loss. For RA patients, chronic inflammation, limited physical activity due to joint pain, and medication side effects further accelerate muscle mass and strength decline, warranting further exploration into muscle wasting in this population.

### Musculoskeletal aging in RA

Musculoskeletal aging in RA involves *myopenia*, *sarcopenia*, and RC. *Myopenia* is muscle loss due to illness at any age, while primary forms of *sarcopenia* are related to aging and secondary forms linked to diseases. RC differs by combining significant muscle loss with a minimal or mild increase in fat accumulation, maintaining stable body weight but increasing mortality. Factors like *Oxidative stress* are recognized as key contributors in accelerating muscle degradation alongside elements like *ACPAs, hormonal shifts*, and *insulin resistance*.

#### Myopenia & Sarcopenia

*Myopenia* refers to clinically significant muscle wasting caused by any illness, regardless of age. In contrast, *sarcopenia* specifically refers to muscle wasting leading to a significant reduction in muscle mass. *Sarcopenia* can be classified into two types: primary and secondary. Primary *sarcopenia* is linked to age-related decreases in muscle mass, strength, and physical performance, illustrating the natural aging process. *Primary sarcopenia* is mainly influenced by aging-related factors like motor unit loss, systemic inflammation, and hormonal changes. In contrast, *secondary sarcopenia* is associated with disease-related declines, such as those from cancer, diabetes, COPD, or heart failure, and involves more severe muscle deterioration due to the underlying disease and its treatments, like chemotherapy or extended bed rest, resulting in a significant decline in muscle function and overall health.^16^

**Fig. 1:**
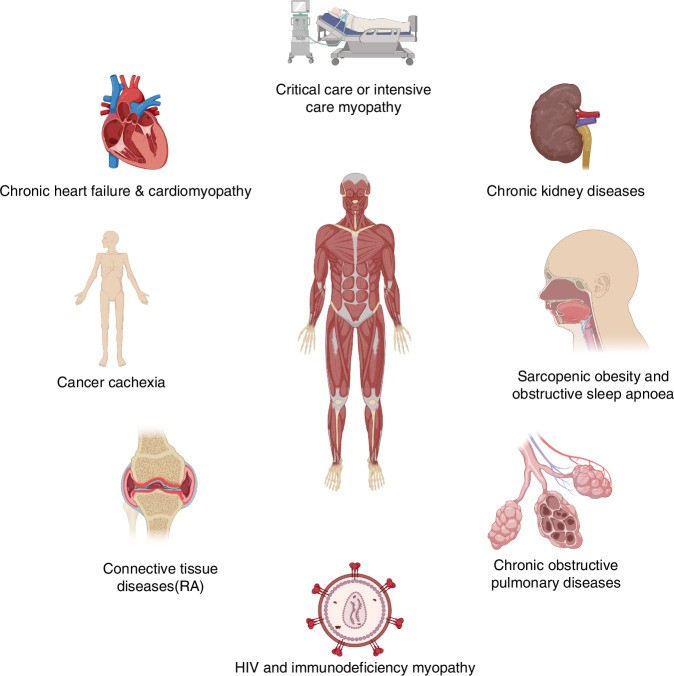
*Myopenia*’s network: linking diseases and morbidity. This diagram illustrates the conditions contributing to musculoskeletal aging in RA, including myopenia, sarcopenia, and rheumatoid cachexia. These conditions, driven by systemic illnesses and inflammation, result in significant muscle loss and increased morbidity. Created in BioRender

*Myopenia* and *secondary sarcopenia* exhibit a non-linear pattern of muscle deterioration due to disease, which is more severe than the linear decline associated with primary *sarcopenia*. Consequently, patients with RA experience significant reductions in muscle mass compared to their age- and sex-matched peers, making *myopenia* an indicator of early, disease-related muscular aging. This condition is particularly relevant to RA and other related diseases (Fig. 1).

### RC and muscle wasting

RC is characterized by a substantial decrease in muscle mass and strength, often accompanied by a minimal or mild increase in fat accumulation.^18^ Unlike the forms of cachexia observed in conditions such as cancer and heart failure, RC typically does not lead to an overall decrease in body weight; the significant muscle mass loss is often offset by an increase in fat mass, resulting in a relatively stable overall body weight.^19^ Moreover, RC differs from primary *sarcopenia* as it tends to manifest at a younger age and involves a more severe loss of muscle mass. The severe loss of muscle mass heightens the risk of cardiovascular and contributes to higher mortality rates,^20^ significantly impairing patients’ quality of life. Importantly, RC also exhibits chronic inflammation, a trait it shares with muscle wasting observed in other chronic conditions.

Muscle wasting, in a broader context, refers to the reduction in muscle mass due to various factors. In earlier research, high-grade inflammation was recognized as a major catalyst for muscle wasting; however, recent findings suggest that inflammation alone does not fully account for the extent of muscle wasting observed in patients with RA. Although additional factors, such as physical activity and nutrition, are recognized to contribute, they also fail to fully explain the significant muscle loss observed. Consequently, researchers are now investigating other potential factors to better understand the mechanisms behind muscle wasting in RA patients.

One emerging factor that may help explain this discrepancy is *Oxidative stress*. Initially, *Oxidative stress* was recognized as a key contributor to muscle wasting in the general population, but its role in RA-related muscle wasting was less clear. Recent studies have confirmed that *Oxidative stress* plays a crucial role in RA-related muscle wasting, exacerbating disease progression by inducing muscle degradation. The mechanisms of how *Oxidative stress* induces muscle wasting will be elucidated in the following chapters. Additionally, other contributing factors, such as *ACPAs*, *immunological* and *hormonal shifts*, *interleukin secretion*, *myostatin*, *intermuscular adipose tissue (IMAT)*, and *insulin resistance*, and their respective roles in muscle wasting, will also be discussed.

## Genetics of RA and muscular aging

The genetic basis of RA plays a key role in disease onset and progression, as well as its impact on muscle loss. Variations in the HLA-DRB1 locus, such as specific alleles tied to the *shared epitope (SE)*, are linked to increased RA risk. Additionally, other gene variants like those in ACTN3 and MSTN may also influence muscle loss in RA through complex polygenic interactions. While no single mutation has been directly linked to muscle wasting, the combined effect of multiple genes likely contributes to the variability in muscle degradation among RA patients. Further research is needed to better understand these genetic influences and their therapeutic implications.

### Genetic factors of RA

The development of RA is heavily influenced by genetic factors, particularly the DRB1 locus within the HLA class II gene complex. Distinct amino acid sequences at positions 70 and 40 in certain DRB1 alleles are linked to RA, corroborating the ‘shared epitope’ hypothesis as a known RA risk factor.^21^ Research has shown that the presence of the DRB1 allele correlates with early disease onset, the occurrence of extra-articular symptoms, and increased radiographic damage.^22^ However, the genetic predisposition in *EORA* remains a subject of conflicting and inconsistent findings.^23^

Notable differences exist in the RA-associated DRB1 alleles between *YORA* and *EORA*, as well as among various ethnic groups. For instance, Gonzalez-Gay et al. conducted research within a Spanish cohort and identified associations between *YORA* and the DRB1/04 allele, and between *EORA* and the DRB1/01 allele.^24^ Their findings revealed that the shared epitope (SE, an amino acid sequence commonly found in certain alleles of the HLA-DRB1 gene), particularly associated with DRB1/04, was significantly more frequent in *YORA* patients, with a frequency of 77.8% compared to 37.1% in *EORA*. This genetic distinction suggests that DRB1/04 plays a stronger role in disease susceptibility and severity among younger RA patients. Furthermore, the study highlighted that *EORA* patients who are seronegative for rheumatoid factor (RF) exhibited a higher frequency of DRB1-13/14 alleles, indicating a potential shared genetic basis with polymyalgia rheumatica (PMR), which was also associated with these alleles. In a parallel study, Kim et al. explored the impact of the HLA-DRB1 and HLA-DQB1 genes on disease susceptibility and severity in both *EORA* and *YORA* patients.^25^ Their findings indicated a lower frequency of alleles encoding the common epitope in *EORA* compared to *YORA*, suggesting a diminished role in susceptibility to *EORA*. Conversely, Hellier et al. reported that HLA-DRB1/04-related alleles are unlikely to be significantly associated with *EORA* susceptibility,^26^ whereas Wu et al. documented a higher prevalence of the DRB1/04 allele in *EORA* patients.^27^ The observed discrepancies may be attributable to variations in the ethnic and racial composition of the study cohorts, potentially affecting the distribution of HLA alleles, necessitating further research to elucidate its role in disease onset and progression across diverse populations.

### Gene variants and polygenic perspective

Numerous gene variants have been proposed as potential contributors to RA, yet evidence establishing strong associations between specific variants and premature muscular aging remains scarce. For instance, although the ACTN3 R577X polymorphism influences human muscle phenotypes, debates persist regarding the ‘favorable’ role of the R or X alleles in aging processes. Moreover, the MSTN K153R variation is recognized for its potential to explain differences in muscle phenotypes among older individuals, but further research with a significantly larger sample size is essential, given the rarity of the ‘unfavorable’ 153 R allele. Despite the current gaps in understanding, ongoing research continues to explore gene variants that may be linked to RA and other autoimmune disorders.

A study by Zhang et al. found associations between the ARNT rs11204735, AHRR rs2292596, and rs2672725 polymorphisms with increased susceptibility to RA and altered AHR methylation levels.^28^ Furthermore, research by Huang et al. has identified crucial genes and pathways potentially related to RA, including STAT4, PTPN22, and TRAF1/C5, which play significant roles in immune regulation and inflammation.^29^ These findings highlight the complex and interconnected nature of RA-related genetic variants, supporting the notion that RA likely results from multiple polygenic traits rather than a single polymorphism.^30^ Wen and Yu discovered significant genetic correlations by identifying shared loci between RA and other autoimmune diseases, including multiple sclerosis, type 1 diabetes, and inflammatory bowel disease, indicating a common genetic basis for these conditions.^31^

### Potential genetic associations between RA and muscle wasting

Existing evidence does not definitively identify a genotype linked to accelerated age-related muscle wasting, recent studies improve our ability to predict such outcomes.^30^

Chronic inflammation, the central pathogenic factor in RA, highlights the importance of understanding IL-6 expression differences at the genetic level. IL-6 plays a crucial role in RA pathogenesis by mediating immune responses, driving chronic inflammation, and promoting bone destruction. Genetic variations, such as single nucleotide polymorphisms (SNPs) in the IL-6 gene promoter region (e.g., -174G/C and -572G/C), are associated with increased RA susceptibility and disease activity.^32^ In RA patients, IL-6 expression is not only elevated systemically but also markedly upregulated in synovial tissues compared to peripheral blood. This localized overexpression contributes to synovial hyperplasia (synovitis) and angiogenesis, which intensify joint damage and have systemic repercussions.^33^ The following section will explore in detail how IL-6 influences muscle wasting mechanisms in RA patient.

### Pathogenesis of musculoskeletal aging in RA

The pathogenesis of musculoskeletal aging in RA involves six key mechanisms, among which oxidative stress—caused by excessive reactive oxygen species (ROS)—drives protein degradation and cellular damage.Anti-citrullinated protein antibodies (*ACPAs*) amplify inflammation, leading to muscle and bone deterioration. Immunological and hormonal alterations in RA patients, particularly immune dysregulation, further exacerbate muscle breakdown. Interleukin-6 (IL-6) activates the JAK-STAT pathway, promoting muscle protein degradation and impairing regeneration. Myostatin inhibits muscle growth by triggering proteolytic pathways, causing atrophy. Lastly, *intermuscular adipose tissue (IMAT)* accumulation and insulin resistance disrupt muscle metabolism, decrease protein synthesis, and accelerate muscle wasting.

#### Oxidative stress

When the production of free radicals, such as *reactive oxygen species (ROS)*, exceeds the capacity of the antioxidant defense system to neutralize them, *Oxidative stress* occurs. Free radicals are highly reactive molecules that can attack lipids, proteins, and DNA, leading to cellular damage. *Oxidative stress* has been shown to play a role in muscle loss in normal aging and metabolic processes. In pathological states, such as RA, this effect is amplified, contributing to more pronounced muscle atrophy.

**Fig. 2:**
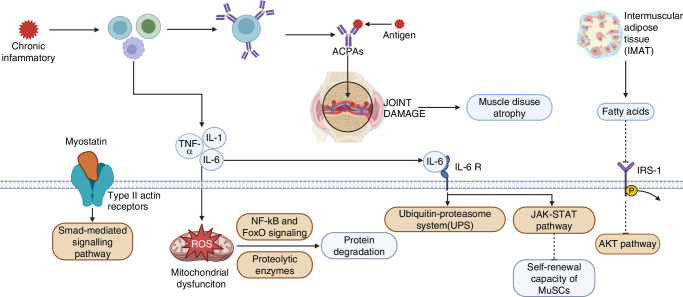
Pathogenesis of musculoskeletal aging in RA. This diagram summarizes the various potential mechanisms that contribute to muscle loss in patients with RA. Created in BioRender

In RA, chronic inflammation is a key pathological feature. In RA patients, he immune system is overactive, leading to the production of numerous inflammatory cytokines like TNF-α, IL-1, and IL-6, which subsequently promote ROS generation (Fig. 2). RA patients exhibit higher ROS levels compared to healthy individuals, alongside a diminished antioxidant capacity. Research indicates that mitochondrial ROS production is elevated in the whole blood and mononuclear cells of RA patients compared to healthy individuals, highlighting *Oxidative stress* as a key factor in RA pathology.^34^

The mechanisms of ROS-induced muscle atrophy are complex. ROS disrupts redox homeostasis, upregulating apoptotic pathways such as the NF-κB and FoxO signaling cascades, which lead to significant protein degradation^35^ (Fig. 2). This oxidative imbalance may also activate proteolytic enzymes, including calpains and caspase-3, thereby further enhancing protein hydrolysis. Moreover, the oxidation of muscle proteins renders them more susceptible to proteolytic damage. In addition to promoting protein degradation, ROS accumulation can inhibit critical signaling pathways that control protein synthesis. Specifically, ROS has been shown to impair mRNA translation at early stages, reducing the ability of muscle satellite cells to activate and infiltrate muscle fibers, ultimately leading to impaired muscle regeneration (Fig. 2). These combined mechanisms contribute to the progressive muscle wasting observed in conditions such as RA.^36^

SIRT1 and HSP play crucial roles in counteracting muscle wasting caused by *Oxidative stress*. They work through different mechanisms to reduce inflammation and oxidative damage, thereby protecting muscle tissues. Firstly, SIRT1, a NAD + -dependent deacetylase, is essential in mitigating *Oxidative stress*. SIRT1 activates the cell’s antioxidant defense system by deacetylating antioxidant enzymes like FOXO1 and FOXO3, which helps reduce the harmful effects of ROS on muscle and joint tissues. Additionally, SIRT1 inhibits pro-inflammatory pathways such as NF-κB, lowering the production of pro-inflammatory cytokines like TNF-α and IL-1β, thus decreasing inflammation-induced muscle degradation.^37^ Secondly, heat shock proteins (HSP), such as HSP-70, have been identified as inhibitors of inflammatory cytokine production, serving as protective factor against *Oxidative stress* to muscle cells.^38^ In contrast, aberrant glycosylation patterns of IgG antibodies are believed to enhance pro-inflammatory activity, thus exacerbating muscle degradation.^39^ A deeper understanding of these mechanisms is crucial for comprehending the full scope of muscle wasting in RA.

### Anti-citrullinated protein antibodies (*ACPAs*)

Two primary subtypes of RA are distinguishable based on the presence or absence of anti-citrullinated protein antibodies (*ACPAs*). *ACPAs* are autoantibodies that target peptides and proteins undergoing citrullination, a post-translational modification that occurs under inflammatory conditions. These autoantibodies are highly specific for RA and are detectable years before the clinical onset of the disease, making them a valuable tool for early diagnosis. Approximately 67% of RA patients are ACPA-positive, establishing *ACPAs* as a crucial diagnostic marker for early, undifferentiated arthritis.^40,41^ The enzyme peptidyl arginine deiminase (PAD) catalyzes citrullination, a calcium-dependent process that converts positively charged arginine into neutral, polar citrulline. The ACPA-positive subtype generally correlates with a more severe clinical course than the ACPA-negative form.^42,43^ This severity is marked by greater bone destruction, driven by increased osteoclast activity. ACPA-positive patients show enhanced osteoclast differentiation and activation, leading to more bone erosion and joint damage, even without obvious inflammation. Although ACPA-negative patients have a milder clinical course, standard therapies such as methotrexate (MTX) and rituximab tend to be less effective in ACPA-negative patients,^44–46^ underscoring the need for further research into the distinct pathophysiological mechanisms of these subtypes. This review will focus particularly on ACPA-positive RA, examining its onset and progression in the contexts of *EORA* and *YORA*.

### Immunological and hormonal shifts

Current insights into the clinical and laboratory differences between *EORA* and *YORA* remain incomplete. However, prevailing theories suggest that the pathogenesis of *EORA* is influenced by immunological and hormonal shifts. As individuals age, there is a notable decrease in protective immune responses alongside an increase in reactivity to autoantigens (Fig. 2). This decline stems from changes in T-cell phenotypes, impaired apoptosis, inadequate antigen presentation, and cytokine imbalances. Age-related thymic involution exacerbates these issues by altering T-cell composition and reducing T-cell proliferation, cytokine production, and antibody synthesis post-vaccination. The decline in protective immune responses, particularly in the ability to clear pathogens and maintain immune tolerance, leads to a loss of self-tolerance. This allows autoreactive T cells to persist and target self-antigens, promoting autoimmune processes such as those seen in *EORA*. The weakened immune surveillance results in an increased reactivity to autoantigens, which is a hallmark of *EORA* compared to *YORA*, contributing to the more aggressive disease progression in elderly patients. Further impairments in age-related self-tolerance mechanisms also weaken the immune response in elderly patients with RA.

### Interleukin secretion and *myopenia*

A study by Straub et al. suggests that serum levels of *DHEA* (Dehydroepiandrosterone, one of the key hormones secreted by the adrenal glands, belonging to the third major category of adrenal steroids) are negatively correlated with IL-6. In healthy individuals, higher levels of *DHEA* are associated with relatively lower levels of IL-6. As age increases, *DHEA* levels gradually decline while IL-6 levels rise, potentially contributing to increased inflammatory responses. The study also indicates that serum *DHEA* levels in RA patients are significantly lower than in healthy individuals. This reduction in *DHEA* levels in RA patients may be linked to certain immunoregulatory dysfunctions associated with chronic inflammatory conditions.^47^ It is hypothesized that increased IL-6 levels contribute to the rapid onset and pronounced acute phase response characteristic of *EORA*. Corroborating this, Punzi et al. found significantly elevated IL-6 levels and acute phase responses in the synovial fluid of *EORA* patients compared to those with *YORA*, highlighting the critical role of IL-6.^33^ Interestingly, no statistical differences were observed in the levels of IL-1 and IL-8 between *EORA* and *YORA* patients. Moreover, Bennett et al. further elaborated that IL-6 plays a pivotal role in the pathophysiology of muscle wasting, particularly in conditions like RA-associated *sarcopenia*.^48^ Chronic elevation of IL-6, along with other pro-inflammatory cytokines, accelerates muscle protein breakdown by upregulating the ubiquitin-proteasome system (UPS), which leads to muscle atrophy^48,49^ (Fig. 2). Additionally, IL-6 disrupts the self-renewal capacity of *muscle stem cells (MuSCs)*, hindering muscle regeneration (Fig. 2). This process is mediated through the JAK-STAT pathway, where chronic activation of STAT3 promotes catabolic processes and muscle loss (Fig. 2). IL-6-induced mitochondrial dysfunction also contributes to reduced muscle contractility and increased *Oxidative stress*, exacerbating muscle fatigue and weakness commonly seen in rheumatoid *sarcopenia*.

Furthermore, Gamerith et al. noted a substantially higher anti-IgG-Fab/free aFab ratio in *YORA* patients than in *EORA* patients. This elevated ratio suggests a greater presence of rheumatoid factor (RF), indicating divergent immunoregulatory mechanisms across different age groups.^50^ Despite this, the differences in RF between *EORA* and *YORA* imply that RF may not play a significant role in the development of *myopenia* in either group. Instead, these findings further support the hypothesis that elevated IL-6 level is the primary driver of muscle wasting in both *EORA* and *YORA*, potentially leading to premature muscle aging. Conversely, the acute onset in *EORA* could lead to sustained muscle damage, thus mimicking premature muscle aging. Comprehensive research is essential to unravel the complex mechanisms contributing to premature muscular aging in RA.

### Myostatin regulation and *myopenia*

Myostatin, scientifically known as *Growth Differentiation Factor-8 (GDF-8)*, is a member of the TGF-β superfamily, specifically belonging to the Growth Differentiation Factor (GDF) subfamily. Furthermore, as a pivotal regulator of muscle growth, myostatin is primarily produced in skeletal muscle and is also present in cardiac muscle and adipose tissue.^51^ Its mechanism of action involves interactions with type II activin receptors, initiating signaling cascades that impede muscle growth and differentiation. Myostatin frequently engages with the Smad-mediated signaling pathway, potentially inducing the expression of atrophy-related ubiquitin ligases such as Atrogin1 and MURF1. Moreover, myostatin inhibits the transcription of genes that promote myogenesis, further restraining muscle growth.^51^ A study by Gonzalez-Ponce et al. revealed that RA patients exhibited significantly higher serum myostatin levels compared to controls, suggesting that elevated myostatin levels (≥ 17 ng/mL) are effective markers for identifying RA complications, *myopenia* and reduced skeletal muscle mass.^6^ Additionally, Lin et al. discovered that high myostatin levels correlated with increased rates of radiographic progression in RA patients. Collectively, RA patients with *myopenia* and elevated myostatin levels exhibited the most substantial radiographic progression, the lowest likelihood of remission, and the poorest long-term outcomes^52^ (Fig. 2). Itoh et al. concluded that Smad3-STAT3 crosstalk plays a significant role in various pathophysiological contexts, such as tumorigenesis, fibrosis, and T cell differentiation.^53^ However, there is currently no relevant research investigating this crosstalk in RC. Given that both STAT3 and Smad3 are downstream mediators in the mechanisms through which IL-6 and myostatin contribute to muscle atrophy, exploring this crosstalk could hold potential research value. Understanding how these two pathways interact might provide new insights into the progression of RC and possibly open avenues for therapeutic interventions.

### *Intermuscular adipose tissue (IMAT)* and insulin resistance

The accumulation of *intermuscular adipose tissue (IMAT)* is a common feature in RA patients and is associated with increased fat mass and decreased muscle mass. IMAT refers to metabolically active fat located within and between skeletal muscle fibers, distinct from subcutaneous and visceral fat. Its accumulation disrupts muscle metabolism by promoting local inflammation and metabolic dysregulation. Unlike subcutaneous and visceral fat, IMAT is embedded within muscle structures and has a direct impact on muscle metabolism and function. IMAT accumulation is accompanied by abnormal release of free fatty acids that enter muscle cells and interfere with insulin signaling by reducing IRS-1(Insulin Receptor Substrate 1) phosphorylation and inhibiting the Akt pathway. The inhibition of IRS-1 phosphorylation disrupts the activation of the Akt pathway, a central component in glucose uptake and metabolism. This impairs the regulation of glucose and amino acid uptake, further reducing muscle protein synthesis and accelerating muscle atrophy.^54^

IMAT also contributes to chronic muscle inflammation by releasing local inflammatory mediators such as MCP-1. This ectopic fat deposition worsens energy and protein metabolism in muscle, contributing to the progression of muscle loss in RA patients^54^ (Fig. 2).

### Unique musculoskeletal aging mechanisms in RA versus other chronic diseases

In chronic diseases, muscle atrophy is a prevalent pathological feature driven by mechanisms such as chronic inflammation, metabolic dysregulation, and impaired signaling pathways. For instance, in diabetes, chronic low-grade systemic inflammation and insulin resistance play central roles, where pro-inflammatory cytokines (e.g., TNF-α, IL-6) suppress muscle protein synthesis and enhance proteolysis. Hyperglycemia and the accumulation of advanced glycation end products (AGEs) further exacerbate oxidative stress and damage to muscle cells.^55^ Similarly, in cancer, muscle atrophy is closely associated with cancer cachexia, a multifactorial syndrome characterized by rapid loss of lean body mass. Pro-inflammatory cytokines within the tumor microenvironment (e.g., IL-6, TNF-α) activate NF-κB and STAT3 signaling pathways, resulting in increased protein degradation, metabolic reprogramming, and mitochondrial dysfunction.^56,57^ These processes underscore the shared yet distinct inflammatory and metabolic drivers of muscle loss in chronic diseases.

In contrast, the mechanisms underlying muscle atrophy in RA are distinct due to the disease’s autoimmune nature and the dual impact of localized joint inflammation and systemic inflammatory effects. RA is characterized by aberrant activation of the immune system, leading to chronic synovitis and systemic inflammation. Elevated levels of inflammatory cytokines such as TNF-α, IL-1, and IL-6 not only mediate joint destruction but also directly contribute to skeletal muscle atrophy through systemic circulation.^58^ These cytokines activate the ubiquitin-proteasome system (UPS) and autophagy-lysosome pathways, promoting proteolysis while concurrently inhibiting anabolic pathways critical for muscle protein synthesis.^48,49^ Furthermore, RA-associated joint pain and functional limitations reduce physical activity and mechanical loading on muscles, exacerbating disuse-induced muscle atrophy. Unlike the metabolic dysregulation driving muscle loss in diabetes or the cachexia-induced systemic catabolism in cancer, muscle atrophy in RA is uniquely characterized by the interplay of local joint inflammation and systemic immune-mediated effects. This multifactorial mechanism highlights the distinct pathological complexity of RA-related muscle loss.

## Clinical features of *myopenia* in RA

*Myopenia* encompasses a spectrum of clinical features, presenting distinct characteristics between *EORA* and *YORA* patients.

### Three distinct clinical patterns of *EORA*

*EORA* can be categorized into three clinical profiles.^59^ The most prevalent profile, observed in 70% of cases, is characterized by joint erosions, RF positivity, and a significantly poorer prognosis compared to *YORA* patients. The second profile, evident in 25% of cases, resembles PMR and is marked by an abrupt onset, inflammation in the proximal limb joints, absence of RF, and generally a better prognosis than that observed in *YORA* patients. Notably, approximately 25% of PMR cases display non-erosive polyarthritis, frequently included in the differential diagnosis.^60^ The third profile is indicative of seronegative *EORA*, characterized by arthritis affecting the metacarpophalangeal (MCP) and proximal interphalangeal (PIP) joints, along with involvement of proximal limb joints. This profile mirrors remitting seronegative symmetrical synovitis with pitting edema (RS3PE) syndrome. It is distinguished by an abrupt onset, spontaneous remission within 3 to 18 months, hand pitting edema, HLA-B27 positivity, and wrist tenosynovitis.^61^

### *EORA* vs *YORA*

*EORA* extends beyond PMR, encompassing conditions such as sarcoidosis, crystal arthritis, and hepatitis C.^62^ *EORA* usually presents acutely with symptoms similar to PMR but has fewer rheumatoid nodules and a lower rate of RF positivity than *YORA*. Patients with *EORA* typically have lower joint scores but show more significant functional impairment, as reflected by elevated Health Assessment Questionnaire disability index (HAQ-DI) scores.

Clinical research^63,64^ has recorded the involvement of both large and small joints at the initial diagnosis of *EORA*. The prevalence of RF and *ACPAs* in *EORA* patients is comparable to or slightly lower than that observed in *YORA* patients. A study focusing on Turkish patients noted a predominant involvement of the shoulder joint in *EORA*, whereas joints such as the PIP joints, elbows, MCP joints, and ankles were more frequently affected in *YORA*.^65^

*EORA* is associated with a lower incidence of RA deformities, less pulmonary involvement, and fewer cases of secondary Sjögren’s syndrome (SjS). Conversely, symptoms such as lymphadenopathy, muscle pain, weight loss, and PMR-like features are more prevalent in *EORA*. The detection rates of serological markers, including RF, anti-Ro, antinuclear antibody, and anti-La, are lower in *EORA* patients. However, van der Heijde et al. identified an increased risk of anemia, elevated erythrocyte sedimentation rate (ESR), and higher C-reactive protein (CRP) levels in this group.^66^

Chen et al. conducted a comparative study revealing that *EORA* patients showed increased IL-6 levels and reduced TNFα levels.^67^ Multivariate analyses indicated that higher IL-1 levels were associated with *ACPAs*, while increased TNFα levels correlated with constitutional symptoms in *EORA* patients. Additionally, *EORA* patients typically presented with an acute onset, constitutional symptoms, and multiple comorbidities, distinguishing them from *YORA* patients.

**Fig. 3:**
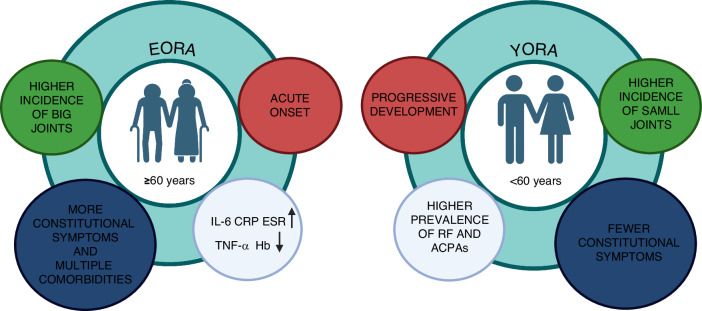
Clinical features of *EORA* vs. *YORA*. The diagram compares the clinical features of elderly-onset RA (EORA) and young-onset RA (YORA), highlighting their differences in joint involvement, onset patterns, and systemic symptoms. Created in BioRender

Lance et al. identified a particularly severe and erosive form of *EORA*, characterized by PMR-like symptoms, acute onset, involvement of both small and large joints, and joint space narrowing.^68^ This aggressive phenotype features rapid disease progression, polyarticular involvement of small joints, joint erosions in the hands and wrists, and early loss of hand function. Remarkably, secondary SjS was noted in 63% of these *EORA* patients, compared to 25% in those diagnosed with *YORA*. (Fig. 3)

Although there are no specific studies currently exploring the differences in myopenia between EORA and YORA, we can still infer some distinctions based on the characteristics of the disease and the differences in the affected populations. EORA patients typically have lower baseline muscle mass and strength due to age-related muscle wasting, which is further exacerbated by RA-associated inflammation, leading to accelerated muscle protein degradation. Additionally, EORA patients are more affected by neuromuscular aging, which significantly contribute to muscle weakness. Reduced physical activity, driven by age-related decline, comorbidities, and joint pain, often resulting in faster progression of myopenia. In contrast, YORA patients typically begin with higher muscle mass and are less affected by neuromuscular aging during the early stages of the disease. However, prolonged chronic inflammation leads to gradual muscle loss, with functional limitations and activity reductions becoming more pronounced over time as the disease progresses. Recognizing these differences is essential for developing strategies.

### *Myopenia* in Chinese RA patients

Previous discussions have highlighted the growing concern of myopenia and muscle wasting in RA patients globally. In the context of RA in China, myopenia has emerged as a significant concern, correlated with physical impairments and joint deterioration.^69^ It is also characterized as a condition linked to body composition, prevalent among elderly Chinese individuals with RA.^4^ Mediation analysis indicated that approximately half of *EORA* patients exhibit *myopenia*, which exacerbates physical dysfunction, highlighting the importance of recognizing cultural and gender-specific nuances within the RA context.^4^

### Laboratory findings of *myopenia* in RA

Research comparing *EORA* with *YORA* consistently demonstrates that *EORA* is characterized by elevated levels of CRP, decreased hemoglobin levels, and an increased ESR.^70^ However, findings regarding the role of *ACPAs* and RF in distinguishing *EORA* from *YORA* are inconclusive. While some studies report a lower frequency of these antibodies in *EORA*, others find similar levels in both groups.^64,66,71^ Serhal et al. observed significantly higher levels of RF and *ACPAs* in *YORA* patients compared to those with *EORA*.^72^ Additionally, Pan et al. reported a positive association between the disease activity score (DAS28-CRP) and *myopenia*, with an adjusted odds ratio (AOR) of 1.56, and found *myopenia* to be independently associated with physical dysfunction, exhibiting an AOR of 2.98 in patients with early RA.^73^ These findings underscore the importance of early and aggressive management of disease activity to prevent *myopenia* and subsequent physical dysfunction in patients with early RA.

### Measurement and assessment of *Myopenia* and muscle wasting in RA

Longitudinal observational studies show that the loss of appendicular skeletal muscle mass due to aging is gradual, with men experiencing approximately a 5% decline after reaching peak muscle mass and women experiencing a slightly lower decline.^74,75^ Muscle loss is often accompanied by an increase in fat mass, leading to a condition known as sarcopenic obesity, which is characterized by the coexistence of *sarcopenia* and obesity, and results in a detrimental body composition^76^ that adversely affects cardio-metabolic health, deteriorates muscle quality, and increases the risk of obstructive sleep apnea, disability, and mortality.^76,77^ Lin et al. found that *myopenia* overlapping with excessive fat presented the worst radiographic scores and highest rates of previous glucocorticoid treatment and hypertension in RA patients with a normal BMI.^78^

The role of BMI in assessing *myopenia* in RA patients remains ambiguous.^5^ Due to the distinct pathophysiological mechanisms involved, RA-associated *myopenia* differs from primary *sarcopenia* in terms of body composition. Unlike primary *sarcopenia*, which is typically characterized by a proportional decrease in both muscle mass and overall body weight, RA patients may present with a normal or even reduced BMI, yet they still experience significant muscle wasting alongside increased fat mass, a condition often termed RC, highlighting the limitations of using BMI as a sole indicator for muscle mass evaluation in RA.^79^ Furthermore, the combined effects of muscle mass reduction, increased fat accumulation and RA-induced joint damage significantly impair patients’ mobility, further exacerbating the progression of *myopenia* and muscle wasting in RA.

Given these differences, traditional measures like BMI may not accurately capture the extent of body composition changes in RA-associated *myopenia*. Instead, more sophisticated methods such as *bioelectrical impedance analysis (BIA)* or *dual-energy X-ray absorptiometry (DXA)* are recommended to assess muscle mass and fat distribution.^80^ BIA, while portable and cost-effective, is susceptible to inaccuracies caused by hydration status and posture, making it more suitable for outpatient and community settings.^81^ In contrast, DXA is considered the gold standard due to its high precision and capability to concurrently evaluate bone density and fat distribution. However, it is limited by factors such as interstitial fluid accumulation during acute inflammation, strict patient positioning requirements, and radiation exposure.^82^ Understanding these limitations is crucial to ensure appropriate application and interpretation of these tools in clinical settings.

### Prognosis of *Myopenia* in RA

Currently, the direct link between *myopenia* and the prognosis of RA remains elusive. Lin et al. identified baseline *myopenia* as an independent risk factor for one-year radiographic progression in RA patients, with an AOR of 2.5.^83^ Research regarding the onset age of RA presents varied outcomes; some studies suggest that *EORA* may have a more favorable prognosis compared to *YORA*, while others report no significant differences or even poorer outcomes. These discrepancies may arise from variations in disease duration, patient selection biases, and differences in seropositivity rates between younger and older patients.

A prospective study by Pease et al. noted that persistent arthritis was observed in 39% of seropositive patients, compared to only 6% of seronegative patients.^84^ Further supporting these findings, Calvo-Alén et al. reported a higher prevalence of swollen joints, greater radiological damage, and increased mortality risks in seropositive patients.^85^ This suggests that RF and *ACPAs* may not serve as reliable prognostic markers for *EORA*. Krams et al. found that *EORA* patients in the ESPOIR cohort exhibited higher erosion rates and HAQ scores, along with lower one-year remission rates compared to *YORA* patients.^86^ However, by the third year, *YORA* patients showed significantly less radiographic progression, lower HAQ scores, and improved remission rates relative to those with *EORA*. In terms of mortality associated with *myopenia* in *EORA*, seropositive patients exhibited significantly higher mortality rates, whereas this trend was not observed in seronegative patients.^70^

Population-based factors such as race and gender should be carefully considered when we discuss the prognosis of *myopenia* in RA. In a clinical study conducted by Baker et al., muscle wasting in RA patients of different races and genders was analyzed.^80^ Their findings revealed that African Americans are at a higher risk of muscle loss, likely due to genetic factors and healthcare disparities. Additionally, males with RA tend to experience more significant muscle wasting and functional decline compared to females. Muscle wasting, or *myopenia*, weakens muscle strength, impairing patients’ physical capacity. As body weight decreases, the mechanical load on bones is also reduced. This leads to a decline in trabecular BMD, cortical bone thickness, and Appendicular Lean Mass Index (ALMI), indicating a deterioration of bone structure, also indirectly indicateing the potential risk of osteoporosis in RA patients.

### Exercise and RA

Studies indicate that individuals with RA exhibit reduced and impaired exercise capacity compared to healthy counterparts. This limitation is attributable to RA-related symptoms such as pain, joint damage, decreased bone density, stiffness, and muscle weakness.^87–89^ Nonetheless, regular physical activity offers numerous benefits for RA patients, including reduced inflammation and enhanced joint function; thus, tailored exercise routines and regimens are recommended.^90^

### Benefits of aerobics and resistance training in RA patients

**Fig. 4:**
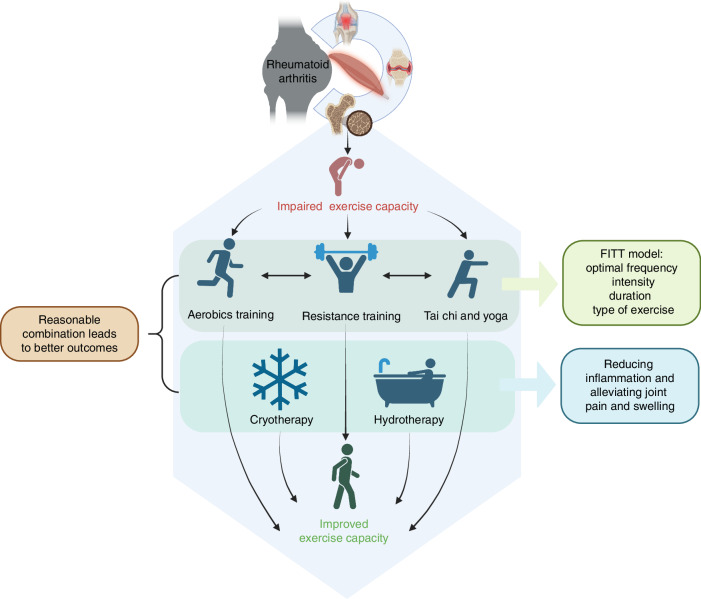
Effects of Exercise Therapy on RA. The diagram illustrates how the reasonable combination of different exercise modalities, guided by the FITT model (Frequency, Intensity, Time, and Type), could restore functions in RA patients. Aerobic training, resistance training, Tai Chi, yoga, and adjunctive therapies like cryotherapy and hydrotherapy work together to improve exercise capacity and alleviate symptoms of RA. Created in BioRender

Structured physical exercise programs are widely recognized for providing significant, lasting benefits to RA patients,^91^ without exacerbating disease activity or causing additional joint damage.^92^ Recent investigations have explored various exercise modalities, including resistance training and aerobic exercises, as well as their combination.^93^ Aerobic activities, such as cycling, swimming, dancing, walking, and running, have been shown to effectively mitigate joint damage in RA patients, provided that the exercise programs adhere to RA-specific guidelines designed to minimize cardiovascular disease risk, maximize quality of life, and maintain daily functional capabilities (Fig. 4).

Cooney et al. explored the impact of exercise interventions on *myopenia* and cachexia, revealing that intensive *progressive resistance training (PRT)* can increase lean body mass, reduce fat mass, enhance strength, and boost overall functionality.^94^ *PRT* is recognized as an effective method for increasing the size and strength of skeletal muscles, and it is deemed safe for RA patients, even at higher intensity levels. Furthermore, *PRT* has been shown to effectively increase mechanical loading on bones, thereby promoting bone mineral density (BMD) enhancement and maintenance. This increased mechanical stimulus plays a critical role in preventing further deterioration of bone structure and mitigating the risk of osteoporosis. For RA patients who are at a heightened risk of osteoporosis due to the prolonged use of corticosteroids, such interventions can delay bone loss and improve skeletal integrity. Resistance training also improves tendon rigidity and strengthens connective tissues, while cyclic loading activities promote cartilage health and enhance joint lubrication. Additionally, when combined with mobility exercises, *PRT* can increase the range of motion for RA patients. Peres et al. also demonstrated that integrating *PRT* exercise programs with cold-water immersion during recovery periods can further improve muscle function, alleviate pain, and enhance overall well-being in RA patients.^95^

According to research by Pedersen et al, skeletal muscle functions as an endocrine organ, releasing myokines such as IL-6 during contraction.^96^ Unlike its previously discussed role as a catalyst in muscle aging, IL-6 released during exercise appears to exhibit anti-inflammatory properties. This is primarily achieved by suppressing TGF-β activity, thus alleviating inflammation and reducing muscle soreness, a process potentially linked to the STAT3-SMAD3 crosstalk. However, the specific effects of muscle-derived IL-6 in RA patients during exercise remain unclear. Further investigation could provide valuable insights into the development of exercise-based therapies for addressing muscle wasting in RA patients.

### Tai Chi and Yoga for RA

The evidence concerning the efficacy of Tai Chi for RA remains ambiguous. A recent Cochrane review concluded that the benefits of Tai Chi on clinical outcomes such as joint pain, activity limitation, and function in RA are uncertain, and neither clear benefits nor adverse effects could be confirmed due to the very low quality of evidence.^97^ Further analysis suggested that while some clinical improvement was noted, it was not statistically significant in terms of pain reduction and disease pattern as assessed using the ACR20 measurement. Improvements in disability and quality of life were observed. Given the low level of evidence in the studies reviewed, a cautious approach to data analysis is advised. The three studies included were found to lack reliability in providing an accurate and comprehensive summary of the effects of Tai Chi on individuals diagnosed with RA.^98^ However, Tai Chi has been shown to significantly improve lower extremity range of motion, particularly in the ankle joint, among individuals with RA.^99^

The evidence for Yoga in treating RA was less convincing than anticipated. A recent case-based review highlighted the potential of Yoga as a supplementary intervention for RA, suggesting it may enhance patients’ quality of life and alleviate disease symptoms.^100^ However, this study suffered from a small sample size, and further research is necessary to substantiate the purported benefits.^100^ More recent studies have shown promising outcomes with Yoga, demonstrating benefits in reducing pain, morning stiffness, and overall disease activity^101^ (Fig. 4).

Although there is no strong evidence to suggest that Tai Chi and yoga have a significant positive impact on the long-term prognosis of RA patients, they do contribute to improving overall function and quality of life. While Tai Chi and yoga may not directly enhance muscle quality as much as strength training or aerobic exercise, they help by improving relaxation, coordination, flexibility, and muscle endurance. Through these mechanisms, they indirectly enhance the efficiency and strength of muscle use, thereby reducing pain, stiffness, and joint dysfunction.

### Symptom control, hydrotherapy, cryotherapy and further research

Exercise has been shown to alleviate RA-associated pain and stiffness, reduce fatigue, and enhance functional capacity and well-being without exacerbating the disease. A study by Al-Qubaeissy et al. indicated that hydrotherapy significantly reduces pain and improves the health status of RA patients.^102^ It is important to note that the pain relief experienced by RA patients from hydrotherapy has only been demonstrated in short-term studies, and its long-term efficacy remains uncertain, requiring further validation. According to the study by Peres et al., cryotherapy, the use of extreme cold to freeze whole-body at –110 °C or local cooling with cold packs or air to reduce inflammation, has shown efficacy in alleviating joint pain and swelling in patients with RA, particularly in those not receiving glucocorticoid therapy.^95^ It is hypothesized that the hypothalamic-pituitary-adrenal (HPA) axis and the sympathetic nervous system (SNS) was active via cold-induced stress during cryotherapy. Notably, patients subjected to cryotherapy exhibited an increase in plasma IL-6 levels, yet their pain scores significantly decreased, suggesting a dissociation between inflammatory markers and clinical symptom improvement.

Nevertheless, further research is essential to determine the optimal frequency, intensity, duration, and type of exercise (*FITT model*) and to assess the effectiveness of combining different types of exercise. For example, *FITT model* optimization, tailored to age, gender, and disease stage, could guide individualized exercise prescriptions. Additionally, more studies are required to explore the best strategies for integrating exercise into patients’ daily routines with varying degrees of disease severity. Different types of exercise, such as low-impact aerobics and resistance training, may provide distinct benefits, and their combination has the potential to enhance therapeutic outcomes. Further research should focus on optimizing exercise therapy by investigating the efficacy of combining these approaches. (Fig. 4)

## Dietary interventions to alleviate muscle wasting in RA patients

It is hypothesized that factors such as dietary habits, inflammation, and physical activity may influence *Oxidative stress* mechanisms, impacting both pro- and anti-oxidative processes to intervene the synthesis of muscle protein. For example, compared to the typical Western diet, which is high in red meat, saturated fats, and refined sugars and is associated with increased inflammation and *Oxidative stress*, the Mediterranean Diet (MD) offers significant benefits. Rich in anti-inflammatory nutrients like fruits, vegetables, whole grains, and fatty fish, the MD helps reduce both inflammation and *Oxidative stress*. Its positive effects are largely due to its high content of antioxidants and omega-3 fatty acids, which balance oxidative and inflammatory processes, potentially alleviating RA symptoms. Additionally, the MD promotes beneficial gut bacteria through its fiber content, further reducing systemic inflammation and *Oxidative stress*.^103^

### Management of frailty and prefrailty in RA *myopenia*

Frailty, associated with decreased muscle strength and elevated inflammatory markers,^104^ contributes to its persistence.^105,106^ Recent findings indicate that frailty and prefrailty occur more frequently in patients with *YORA* than previously recognized.^107^ As frailty becomes more prevalent with age, timely management of frailty is critical for addressing *myopenia*. Future research could focus on assessing frailty in a large cohort to determine whether RA patients who achieve sustained remission with early treatment and avoid joint damage exhibit lower frailty scores compared to those who receive less intensive treatment.

### *Myopenia*, heart failure and premature myocardial aging in RA

RA, like many chronic autoimmune diseases, is associated with significantly elevated cardiovascular morbidity and mortality.^108–112^ Notably, the cardiovascular risk for RA patients is comparable to that of individuals with type 2 diabetes mellitus.^113,114^ This risk is influenced by common factors such as hypertension, obesity, metabolic dysfunction-associated fatty liver disease (MAFLD), dyslipidemia, and hyperglycemia, as well as RA-specific factors including elevated systemic inflammation.^111,115–117^

Additionally, the association between RA and metabolic syndrome further complicates the cardiovascular risk profile. Kerekes et al. highlighted that RA patients often exhibit components of metabolic syndrome, including central obesity, dyslipidemia, insulin resistance, and hypertension, which all contribute to an increased cardiovascular disease (CVD) risk.^79^ Unlike in the general population, RA patients present a “lipid paradox,” where lower levels of total cholesterol and LDL cholesterol are paradoxically associated with higher cardiovascular risk due to the pro-inflammatory state that drives atherosclerosis. Furthermore, RA-specific alterations in adipokine profiles, such as elevated levels of pro-inflammatory leptin and resistin, exacerbate metabolic dysregulation and contribute to both metabolic syndrome and cardiovascular complications.

Heart failure (HF), sometimes termed *myopenia* in cardiac muscle, is linked to increased inflammation and prevalent cardiovascular risks.^118,119^ Pro-inflammatory cytokines in HF contribute to myocardial damage, cardiac muscle *myopenia*, as well as other pathological outcomes via mechanisms such as arterial stiffness and endothelial dysfunction.^120–126^ The systemic inflammation observed in RA exacerbates these processes, accelerating the development of both systolic and diastolic dysfunction as well as left ventricular concentric remodeling. This chronic inflammatory burden not only directly contributes to myocardial aging but also interacts with metabolic syndrome components, such as insulin resistance and dyslipidemia, worsening cardiovascular outcomes.^5^

Numerous studies have assessed the likelihood of HF in RA patients, revealing that HF can develop independently of traditional risk factors among this demographic.^120,124,127,128^ Ischemic heart disease, a common cause of HF, does not fully account for the elevated HF risk observed in RA. Research indicates that non-ischemic HF risks in RA are closely associated with the severity of the disease and can manifest early.^124^ A recent Danish cohort study^129^ identified that RA patients exhibit a 30% greater rate of hospitalization for HF compared to the general population. These findings suggest that RA may significantly contribute to the risk of HF and accelerate cardiac aging. RA patients frequently exhibit elevated levels of cardiac biomarkers, such as troponins and pro-B-type natriuretic peptides, which are critical indicators of heart disease and HF prognosis.^130^ Thus, the systemic inflammation associated with RA may independently elevate the risk of HF and accelerate myocardial aging, irrespective of traditional risk factors. It is likely that the increased risk of HF in RA patients involves systolic and diastolic dysfunction, as well as left ventricular concentric remodeling.^120,121,124,128^

Given the complex interaction between RA, metabolic syndrome, and cardiovascular health, research indicates that the increased risk of HF in RA patients involves not only systolic and diastolic dysfunction but also the synergistic effects of systemic inflammation and metabolic dysregulation. The interplay between these factors leads to a higher incidence of atherosclerosis, myocardial fibrosis, and ultimately heart failure.

Ongoing and future research should investigate the potential benefits of comprehensive heart failure screening in this population. Are there specific biomarkers that could serve as early warning signals for cardiovascular diseases associated with RA? Does the detailed relationship between systemic inflammation and myocardial remodeling need to be confirmed through long-term follow-up studies? Should routine screening for heart failure be considered in RA patients, and what would be the cost-effectiveness of such an approach?

### *Myopenia* and pharmacological intervention in RA

The age of onset and disease progression in RA are crucial in determining the optimal strategies to delay and treat *myopenia*. Although limited data exist on the effectiveness of pharmacological treatments such as synthetic *DMARD*s and biologics in managing *myopenia*, the European League Against Rheumatism supports and advocates for their use. This approach utilizes the anti-inflammatory properties of *DMARD*s to mitigate *myopenia*, delay muscle aging, and reduce cardiovascular morbidity and mortality associated with RA.^112,113^ Additionally, *DMARD*s are postulated to improve joint health and function, potentially allowing for higher levels of physical activity, which may subsequently decrease additional risk factors such as hypertension and diabetes.^131,132^

### Studies analysing DMARDs

A comprehensive review of 12 studies revealed that patients who received *DMARD* treatments following more severe disease progression exhibited an increased likelihood of radiographic joint space narrowing and bone erosions.^133^ These findings strongly correlate with the presentation of *myopenia* in RA, as corroborated by a recent cross-sectional study.^69^ Moreover, a study by Masoud et al. highlighted that synthetic *DMARD*s and biologics may reduce the risk of sudden cardiac mortality among RA patients.^131^ Similarly, Wu et al. discovered that RA patients treated with a combination of at least one *DMARD* and statins experienced a reduction in cardiovascular-related mortality and significant decreases in disease activity.^134^ Given the efficacy of *DMARD*s as a treatment and the adverse outcomes associated with suboptimal RA management—primarily premature bone erosions and poorer functional outcomes—prompt intervention is imperative.^135^ Consequently, a rheumatologist’s assessment to confirm an RA diagnosis and initiate *DMARD*-based treatment promptly is crucial for any patient presenting with unexplained new-onset polyarthritis.

### Oral corticosteroids and pharmacological therapies

**Fig. 5:**
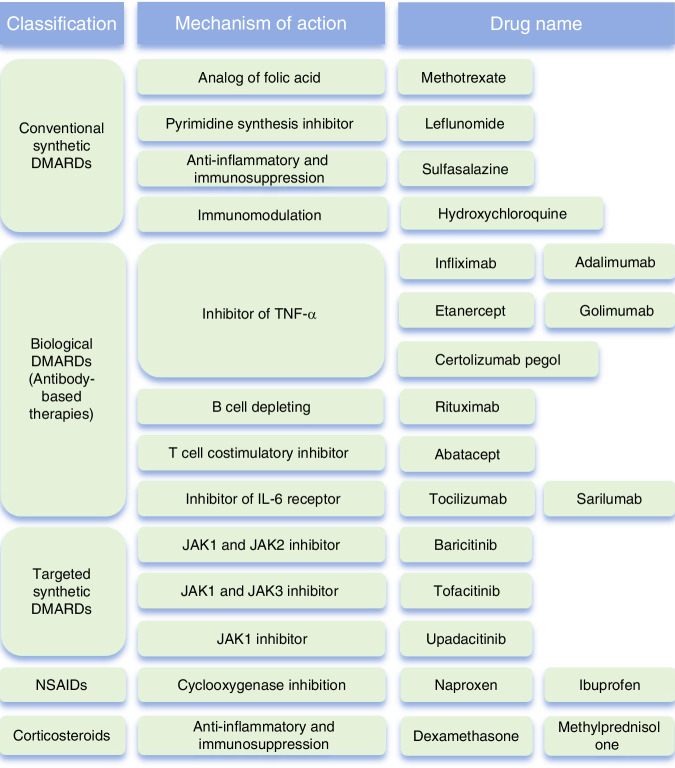
Contemporary pharmacological therapies for RA. This figure outlines classification, mechanism of action, and names of therrapeutic agents of RA

Oral corticosteroids are anti-inflammatory medications that can modify the progression of RA disease,^136^ potentially delaying the onset of *myopenia* and promoting healthy musculoskeletal aging. However, these benefits must be weighed against the significant risk of osteoporosis. The primary treatment focuses on managing symptoms with practical measures to alleviate joint stiffness, pain, and fatigue by reducing systemic inflammation. Based on the articles by Guo et al. and Ben et al. and in conjunction with 2021 American College of Rheumatology Guideline for the Treatment of Rheumatoid Arthritis, we summarized the available options for contemporary pharmacological therapies for RA^137–139^(Fig. 5).

### Tailored treatment approaches for RA

The primary objectives of RA treatment are to manage RA disease progression, reduce radiographic and structural damage progression, and preserve functional capacity.^139,140^ As previously mentioned, the treatment approach for *EORA* should closely align with that for *YORA*. Thus, the ultimate goal should be achieving either remission or low disease activity, adhering to treat-to-target principles.

### Pharmacological considerations and treatment disparities

While *DMARD*s used for *YORA* are generally applicable to *EORA*, it is crucial to consider the altered pharmacokinetics and pharmacodynamics in older patients, necessitating vigilant monitoring for side effects.^141^ Patients with *EORA* often contend with a higher prevalence of comorbidities and engage in polypharmacy, thereby increasing the risk of drug-drug interactions and potential adverse effects.^142^ Treatment strategies for *EORA* tend to be less aggressive and more variable compared to those for *YORA*. Data from the CORONA database indicate that *EORA* patients receive less intensive treatment than their age- and sex-matched *YORA* counterparts, despite similar levels of disease activity and severity.^141^

According to the same database, MTX usage was slightly higher among *EORA* patients (63.9%) compared to *YORA* patients (59.6%), although the average MTX dosage was higher among *YORA* patients.^141,143^ Notably, *EORA* patients were less likely to use multiple conventional or biological *DMARD*s compared to *YORA* patients. The incidence of drug-related toxicity was comparable in both groups, though MTX toxicity occurred more frequently in *YORA* patients. These findings suggest that age may influence the severity of the disease and the range of treatment options, with *EORA* patients often receiving less aggressive therapy despite having similar disease durations, activities, and severities as *YORA* patients.

Swiss registry data revealed that first-line corticosteroid use was significantly higher among *EORA* patients, along with a lower follow-up rate for biological drug use.^143^ In a study by Genevay et al. involving 1 571 RA patients treated with anti-TNFα drugs, withdrawal rates and changes in Disease Activity Score (DAS28) were consistent across groups after two years.^144^ However, despite clinical improvements, *EORA* patients exhibited significantly less enhancement in HAQ scores. Moreover, TNF inhibitors were shown to be less effective in *EORA* patients compared to *YORA* patients, as evidenced by lesser improvements in HAQ scores.^145^

### Conclusive analysis of *DMARD*s in *EORA* and *YORA*

Tocilizumab, abatacept, rituximab, and tofacitinib exhibit limited evidence of effectiveness in treating *EORA*. Specifically, tocilizumab demonstrates lower efficacy in *EORA* compared to *YORA*, although drug retention rates and discontinuation due to side effects are comparable in both groups.^146^ Additionally, no data are available on the effectiveness of abatacept in *EORA*, but tofacitinib has been shown to be equally effective in both *EORA* and *YORA* according to randomized controlled trials.^147^

A recent study on *myopenia* in *EORA* and *YORA* has revealed several distinct characteristics of *EORA*: a more balanced sex distribution, a higher incidence of acute onset with systemic symptoms, increased involvement of large joints, and reduced RF positivity.^148^ *EORA* patients are generally diagnosed earlier, exhibit less erosive disease, and use *DMARD*s less frequently than *YORA* patients. Further research is imperative to explore the impact of *myopenia* on the prognosis of RA, particularly focusing on *secondary sarcopenia*, cachexia, and frailty in older adults. Understanding these factors is crucial for assessing their influence on RA outcomes, while also considering the side effects of *DMARD*s in both *EORA* and *YORA*.

### Therapeutic approaches to mitigate muscle loss in RA

Besides traditional pharmacological intervention and their tailored treatment approaches to RA, several drug classes may mitigate muscle loss through various mechanisms.

Anti-inflammatory agents: Chronic inflammation significantly contributes to muscle wasting in RA. TNF-α inhibitors reduce systemic inflammation by blocking TNF-α, which activates the NF-κB pathway and upregulates the ubiquitin-proteasome system, leading to muscle protein degradation. By inhibiting TNF-α, these agents help preserve muscle mass. Similarly, IL-6 inhibitors target IL-6, which activates the JAK/STAT pathway, promoting muscle catabolism.^149^ JAK inhibitors also block this pathway, may further mitigating muscle degradation and supporting muscle health.

Anabolic therapies: In addition to controlling inflammation, anabolic agents such as selective androgen receptor modulators (SARMs) and myostatin inhibitors have emerged as promising treatments for muscle loss.^150^ SARMs stimulate muscle protein synthesis and prevent muscle atrophy by selectively activating androgen receptors, while myostatin inhibitors reduce muscle wasting by blocking myostatin, a negative regulator of muscle growth.

Nutritional and metabolic modulators: Nutritional supplements like vitamin D, omega-3 fatty acids, and branched-chain amino acids (BCAAs) play supportive roles in preserving muscle mass. Vitamin D enhances muscle function and reduces inflammation, improve muscle protein synthesis, and BCAAs help to decrease muscle breakdown and support recovery.^151–154^

Together, these therapeutic strategies represent promising avenues for addressing muscle loss in RA. They not only aim to control the underlying inflammation but also target the anabolic and metabolic processes critical for maintaining muscle mass and function.

### Integrated management of RA: combining pharmacologic and non-pharmacologic approaches

Pharmacologic intervention remains the cornerstone of RA management, aiming to control inflammation, slow disease progression, and prevent joint damage while preserving functional capacity and quality of life. DMARDs are the foundation, effectively suppressing systemic inflammation, improving joint function, and mitigating RA-associated complications.^131,134^ Additionally, the previously mentioned drugs with potential to mitigate muscle loss should also be considered. However, these treatments carry risks such as gastrointestinal issues, liver dysfunction, infection susceptibility, and osteoporosis, necessitating regular monitoring and individualized adjustments to balance efficacy and safety.

Non-pharmacologic interventions complement pharmacologic therapy by addressing broader health aspects. Personalized exercise programs based on the FITT principle (Frequency, Intensity, Time, Type) enhance joint mobility, muscle strength, and bone health while reducing inflammation. Dietary interventions, such as the Mediterranean diet rich in omega-3 fatty acids and antioxidants, support joint health and reduce systemic inflammation.^103^ Psychological support, through cognitive behavioral therapy (CBT) and patient groups, addresses depression and anxiety, improving adherence and overall well-being.^155^

Due to variations in patients’ age, disease duration, and comorbidities, traditional fixed-dose pharmacological treatments often fail to achieve optimal efficacy. Personalized treatment plans tailored to the individual patient’s condition can effectively improve outcomes. By combining pharmacologic and non-pharmacologic strategies, RA management achieves comprehensive disease control, improving both clinical outcomes and patient quality of life. Pharmacologic treatments provide foundational inflammation control, while non-pharmacologic approaches enhance holistic, long-term care.

## Conclusion

The intersection of *myopenia* and accelerated musculoskeletal aging in RA represents a multifaceted area of research, highlighting both the urgency and potential for optimizing patient outcomes through targeted care. This review has examined the pathophysiological mechanisms of muscle wasting in RA, underscoring inflammation, *Oxidative stress*, cytokine activity and hormonal and genetic factors as central contributors. These insights offer a robust foundation for developing targeted interventions that address both muscle degradation and the overall disease trajectory, thereby improving physical function and quality of life for RA patients.

To address the specific complexities of RA-related muscle wasting, a multidisciplinary approach involving pharmacological treatment, personalized exercise interventions, nutritional support, and psychological therapy has been shown to effectively mitigate muscle wasting and improve functional outcomes and quality of life in patients with RA. Early detection of *myopenia* combined with a tailored management strategy encompassing pharmacological treatments, individualized exercise regimens designed by physical therapists, psychological support from mental health professionals and dietary modifications may alleviate muscle loss, reduce frailty, and mitigate risks associated with sarcopenic obesity. Specific pharmacological treatments such as cytokine inhibitors, muscle-enhancing agents, and emerging biologic therapies should be considered for their potential to improve muscle mass and function. The integration of these strategies in clinical practice could significantly delay the progression of muscle wasting and enhance patient resilience, fostering improved physical functionality and lower mortality risks associated with RA.

Future research directions should focus on expanding the understanding of RA-specific muscle loss mechanisms within a collaborative framework. This includes further investigation of pharmacological approaches such as *DMARD*s and biologics, which show potential in mitigating inflammation and slowing muscle decline. Additionally, personalized treatment options, taking into account individual inflammation levels, disease severity, and comorbidities (e.g., age, obesity, metabolic conditions), are likely to optimize care and improve outcomes for RA-associated muscle deterioration.

This review aims to inspire ongoing exploration into RA-related *myopenia* and its implications across diverse RA populations. By advancing research in this critical area, we can contribute to a holistic and patient-centered approach in managing chronic inflammatory diseases, fostering joint health, muscular resilience, and overall physical well-being.
